# Development and Psychometric Validation of a Knowledge Assessment Tool on Radiotherapy Resistance Among Oncologists in Iraq

**DOI:** 10.1007/s13187-025-02687-y

**Published:** 2025-07-24

**Authors:** Bassam Abdul Rasool Hassan, Ali Haider Mohammed, Ahmed Zuhair Abdulhameed Alsammarraie, Musaab Kadhim Alabboodi

**Affiliations:** 1 Department of Pharmacy, Al Rafidain University College, Baghdad, Iraq; 2 School of Pharmacy, Monash University Malaysia, Jalan Lagoon Selatan, 47500 Bandar Sunway, Selangor, Malaysia; 3 Medical Oncology Department, Oncology Teaching Hospital Baghdad, Baghdad, Iraq; 4 Medical Oncology Department, Alamal National Hospital for Cancer Treatment, Baghdad, Iraq

**Keywords:** Radiotherapy resistance, Knowledge, Oncologists, Questionnaire development, Iraq

## Abstract

Radiotherapy resistance (RTR) is a significant barrier in oncology, affecting treatment outcomes and clinical decision-making. Despite its importance, no standardized tool exists to measure oncologists' knowledge of RTR. This study aimed to develop and validate a comprehensive questionnaire to assess oncologists' knowledge of RTR. A 29-item questionnaire was developed following a multi-phase process. An initial pool of 45 items was refined through content and face validity assessments by a panel of 5 experts, leading to item revisions and deletions. Data were collected from 400 oncologists in Iraq. Exploratory Factor Analysis (EFA) and Confirmatory Factor Analysis (CFA) were conducted to establish construct validity, while Cronbach's alpha, Composite Reliability (CR), and Item Response Theory (IRT) analysis were used to assess internal consistency and item performance. EFA confirmed the eight-domain structure of the questionnaire, with factor loadings exceeding 0.70 for all items. CFA demonstrated good model fit, with indices of CFI = 0.92, TLI = 0.91, and RMSEA = 0.06. Internal consistency was high, with a Cronbach's alpha of 0.93 and a CR of 0.94 for the overall questionnaire. IRT analysis revealed that all items had acceptable difficulty, discrimination, and guessing parameters, indicating that the items effectively differentiate oncologists with varying levels of knowledge. The final 29-item questionnaire covered key RTR domains, including Conventional and Combined Therapies, Radioresistance, and Sensitivity and Resistance. The validated 29-item knowledge questionnaire demonstrated strong psychometric properties, making it a reliable tool for assessing oncologists' knowledge of RTR. This tool can support oncology education, training, and research. Future studies should focus on cross-cultural validation, test–retest reliability, and examining how RTR knowledge influences clinical decision-making. This tool is especially relevant for improving oncology training and clinical decision-making in Iraq, where systemic barriers such as limited equipment, training gaps, and uneven access to radiosensitizers challenge cancer care.

## Introduction

Radiotherapy (RT) remains one of the most essential and widely used cancer treatment modalities, with approximately 50% of all cancer patients receiving RT as part of their therapeutic regimen [1]. RT plays a pivotal role in curative, palliative, and adjuvant treatment, enhancing patient survival and quality of life [2]. Despite its significant contribution to cancer control, radiotherapy resistance (RTR) has emerged as a critical barrier to treatment success. RTR occurs when cancer cells develop the ability to survive and proliferate despite exposure to radiation doses that would typically induce cell death [3, 4]. This resistance compromises treatment efficacy, leading to disease progression, recurrence, and poorer survival outcomes. The growing recognition of RTR as a major clinical challenge underscores the need for increased attention to its underlying mechanisms and its impact on oncological practice [5, 6]. Radiotherapy in Iraq is delivered through a limited number of public cancer centers equipped with linear accelerators and cobalt-60 machines. However, disparities exist in access and quality across provinces. Many oncologists report constraints such as outdated equipment, inconsistent maintenance, and limited access to radiosensitizing agents. National treatment protocols are often adapted from international guidelines but lack uniform implementation due to infrastructural and logistical limitations. These systemic barriers necessitate equipping oncologists with comprehensive knowledge of RTR to support adaptive decision-making in challenging clinical contexts [5].

RTR arises from a complex interplay of biological and molecular factors, including genetic mutations, enhanced DNA repair, survival signaling pathways, and changes in the tumor microenvironment [4]. Notably, hypoxia and cancer stem cells are key contributors, promoting resistance by reducing radiation-induced DNA damage and enhancing cellular repair capacity. These mechanisms underscore the importance of oncologists staying informed, as knowledge of RTR can influence clinical decisions such as dose adjustments, use of radiosensitizers, and combination therapies. [7, 8].

Despite the growing body of research on RTR mechanisms, there is limited understanding of the extent to which oncologists are aware of and apply this knowledge in their clinical practice [9]. Studies have highlighted the importance of assessing the knowledge and perceptions of healthcare professionals as a means to identify gaps and improve educational initiatives. However, existing literature indicates that there is no standardized instrument to measure oncologists'knowledge of RTR, making it difficult to assess the readiness of healthcare systems to adopt evidence-based practices in this area. [9, 10].

The development of a validated questionnaire to assess oncologists'knowledge of RTR is therefore crucial. Such a tool can support continuous professional development, enable targeted training programs, and ultimately improve the quality of oncology care [11]. Previous studies on healthcare professionals'knowledge have demonstrated the effectiveness of structured assessments in identifying educational needs and informing competency-based training [11–13]. By developing and validating a questionnaire focused on RTR, this study seeks to provide a systematic approach to identifying knowledge gaps and ensuring that oncologists are well-equipped to address one of the most pressing challenges in cancer treatment.

Iraq presents a critical setting for this research due to its increasing cancer burden, limited healthcare infrastructure, and underdeveloped oncology training programs. According to WHO estimates, cancer is among the leading causes of death in Iraq, with rising incidence attributed to environmental pollution, post-conflict exposures, and lack of preventive screening programs [11]. Iraq has experienced a notable increase in cancer incidence over the past two decades, with breast, lung, and gastrointestinal cancers among the most prevalent. It is reported that cancer accounts for approximately 10% of all deaths in Iraq, with limited access to early detection and specialized care contributing to poor outcomes [9, 11]. These trends reflect the urgent need for enhanced oncologist training and tools to address emerging challenges such as radiotherapy resistance. Despite these challenges, radiotherapy remains a central modality in cancer treatment. However, national data reveal significant variability in oncologists’ access to up-to-date knowledge and continuing education, especially concerning treatment resistance. Moreover, Iraq's oncology education system lacks standardized modules on radiotherapy resistance, underscoring the need for targeted assessment tools. Conducting this study in Iraq allows us to address a pressing educational gap and enhance the delivery of evidence-based cancer care in resource-constrained settings [11].

Therefore, this study aims to develop and psychometrically validate a comprehensive questionnaire to assess oncologists'knowledge of RTR. The ultimate goal is to create a robust, evidence-based assessment tool that can be used in both educational and clinical settings to support oncologists'professional development and enhance patient outcomes. By addressing the knowledge gaps related to RTR, this study aims to facilitate the adoption of more effective treatment strategies, improve patient care, and reduce the negative impact of radiotherapy resistance on cancer outcomes.

## Methods

### Methods

#### Study Design and Setting

This study employed a cross-sectional design to develop and validate a questionnaire aimed at assessing oncologists'knowledge of radiotherapy resistance (RTR). The study was conducted in oncology centers and hospitals across Iraq. Iraq was chosen as the study setting due to its robust oncology infrastructure, where oncologists frequently encounter complex cancer cases, including those related to radiotherapy resistance. The diverse clinical experiences of Iraqi oncologists provide a suitable context for validating the questionnaire, ensuring that the tool can later be adapted for use in both national and international oncology care settings. Iraq's central role in regional healthcare, combined with its growing focus on oncology education and training, further supports its selection as an optimal site for this study. In Iraq, oncologists typically complete medical school followed by board certification in oncology or radiation oncology, offered through institutions such as the Iraqi Board of Medical Specializations. While training includes foundational knowledge of radiotherapy, emerging topics such as radiotherapy resistance are rarely covered in depth. Continuing medical education on RTR is largely self-initiated and hindered by limited access to international conferences and specialized workshops. These educational gaps underscore the necessity of developing assessment tools to support knowledge evaluation and curriculum reform in Iraqi oncology training.

#### Questionnaire Development

The development of the questionnaire followed a structured and systematic process aimed at ensuring its validity, reliability, and practical application. The initial step involved a comprehensive review of the existing literature on RTR, including theoretical frameworks, research studies, and best practices in radiotherapy. This review highlighted key concepts and essential areas of knowledge required for oncologists to effectively understand and address RTR. Based on this, the content of the questionnaire was organized into eight critical knowledge domains: conventional and combined cancer therapies, radioresistance and its limitations, mechanisms of radiation therapy, sensitivity and resistance in cancer cells, radiosensitizers and resistance, chemotherapy and radiation resistance, risk factors, and mechanisms of action of resistance as shown in the supplementary appendix [14–19].

Each of these domains reflects core areas of knowledge that oncologists must master to improve patient care and treatment outcomes. For instance, understanding conventional and combined cancer therapies ensures that oncologists are aware of when to combine radiotherapy with other modalities such as chemotherapy. The domain on radioresistance and its limitations focuses on the basic definition and types of resistance oncologists encounter, while mechanisms of radiation therapy address the role of DNA strand breaks and the impact of ionizing radiation on cancer cells. The sensitivity and resistance in cancer cells domain ensures oncologists understand how tumor type and genetic pathways, such as the p53 pathway, affect response to treatment. The domains on radiosensitizers and resistance, as well as chemotherapy and radiation resistance, introduce oncologists to pharmacological agents and their role in enhancing or reducing the effectiveness of radiation therapy. Lastly, the risk factors and mechanisms of action of resistance domains emphasize how tumor biology, patient history, and clinical conditions influence resistance.

An initial pool of 45 items was generated, with items framed as statements or questions that required a response of “Yes,” “No,” or “Unsure”. This format was chosen to simplify response collection and enhance clarity. Each item was designed to align with a specific knowledge domain and to assess a key aspect of oncologists'understanding of RTR.

#### Content and Face Validity

To ensure the clarity, relevance, and comprehensiveness of the questionnaire, content and face validity were established through a multi-stage review process. A panel of five subject-matter experts (SMEs), consisting of experienced oncologists, radiotherapists, and psychometricians, independently reviewed the items for relevance, clarity, and appropriateness. The experts rated each item on a 4-point Likert scale (1 = not relevant, 2 = somewhat relevant, 3 = quite relevant, 4 = highly relevant).

The Content Validity Index (CVI) was calculated as the proportion of experts rating each item as 3 or 4. Items with a CVI below 0.80 were revised or eliminated. The Modified Kappa Statistic (k)* was also calculated to assess inter-rater agreement beyond chance, and items with k* values below 0.74 were further reviewed [20]. As a result of this process, 12 items were revised, 5 items were removed, and 3 new items were added to improve clarity and coverage of the eight knowledge domains. Following these adjustments, face validation was conducted with 10 practicing oncologists, who reviewed the revised questionnaire to assess item clarity, readability, and relevance. Their feedback led to 7 items revised and 14 items removed, resulting in a final version of the questionnaire containing 29 items.

#### Construct Validity

To assess construct validity, Exploratory Factor Analysis (EFA) and Confirmatory Factor Analysis (CFA) were conducted. For the EFA, a sample of 200 oncologists was recruited from cancer centers and hospitals in Iraq. The suitability of the data for factor analysis was verified using the Kaiser–Meyer–Olkin (KMO) measure of sampling adequacy and Bartlett’s test of sphericity [21]. The KMO value was 0.72, and Bartlett’s test was statistically significant (p < 0.001), indicating that the data were suitable for factor analysis. Principal component analysis with varimax rotation was used to extract factors. Items with communalities below 0.50 or cross-loadings on multiple factors were initially flagged for removal. However, all 29 items demonstrated acceptable communalities, so no items were removed during this process.

To further verify the factor structure, a Confirmatory Factor Analysis (CFA) was conducted on an independent sample of 200 oncologists. The CFA was conducted using AMOS version 26, and model fit was assessed using fit indices, including the Comparative Fit Index (CFI), Tucker-Lewis Index (TLI), and Root Mean Square Error of Approximation (RMSEA) [21]. The model fit indices indicated an acceptable model fit with CFI = 0.92, TLI = 0.91, and RMSEA = 0.06. No items were removed following the CFA, and the final version of the questionnaire retained all 29 knowledge items.

#### Item Response Theory (IRT) Analysis

To further assess the psychometric properties of the 29-item questionnaire, Item Response Theory (IRT) analysis was performed using a 3-parameter logistic (3PL) model [22]. This model was selected as it evaluates item difficulty (b), item discrimination (a), and guessing (c) parameters. The use of IRT offers deeper insights into how well individual items differentiate between respondents with varying levels of knowledge.

IRT analysis was conducted using IRTPRO software. The difficulty (b), discrimination (a), and guessing (c) parameters were estimated for each item. Items with low discrimination (a < 0.30) or excessively high guessing parameters (c > 0.25) were flagged for further review [22]. The item characteristic curves (ICCs) were also plotted for each item, allowing visual inspection of the items’ ability to discriminate between respondents with low, medium, and high knowledge of RTR. All items demonstrated acceptable discrimination and difficulty, and no items were removed as a result of IRT analysis.

#### Reliability Assessment

To ensure the reliability of the questionnaire, Cronbach’s alpha was calculated for each of the eight knowledge domains. A threshold of 0.70 was set for acceptable internal consistency [23]. Composite Reliability (CR) was also calculated to provide an alternative measure of construct reliability [24]. Both Cronbach’s alpha and CR exceeded 0.70 for all domains, indicating strong internal consistency.

#### Data Collection and Participants

A stratified purposive sampling method was employed to ensure representation of oncologists from diverse institutional backgrounds, including tertiary cancer centers, public teaching hospitals, and specialized oncology units across multiple provinces in Iraq. This approach aimed to capture variability in clinical experience, educational exposure, and access to radiotherapy resources. Invitations were disseminated via professional channels such as the Iraqi Oncology Society, departmental administrative contacts, and professional mailing lists. Out of 470 oncologists invited to participate, 400 completed the questionnaire, resulting in a response rate of 85.1%.

Inclusion criteria required participants to be board-certified or licensed oncologists with a minimum of two years of direct clinical experience, specifically involved in radiotherapy treatment planning, delivery, or decision-making. Exclusion criteria encompassed general practitioners, medical interns, or those not actively practicing in oncology.

While it was recognized the potential for self-selection bias due to voluntary participation, several measures were taken to mitigate this. The recruitment strategy targeted a wide range of institutions varying in size, geographic location, and resource availability. Moreover, descriptive analyses of demographic and professional variables revealed no significant discrepancies between the invited and participating oncologists based on institutional role, years of experience, or gender, suggesting that the sample is broadly representative of the national oncology workforce. These measures enhance the external validity and applicability of the findings to the broader oncologist population in Iraq.

#### Scoring Method and Knowledge Classification

A scoring system was established to classify oncologists’ knowledge of RTR. Each correct answer was assigned 1 point, while incorrect or “unsure” responses were assigned 0 points. The total knowledge score was calculated as the sum of the points for the 29 knowledge items, with a maximum possible score of 29 points. Knowledge levels were classified as follows: poor knowledge- total score less than 50% (0–14 points); moderate knowledge-total score between 50 and 74% (15–21 points); and good knowledge- total score of 75% or higher (22–29 points) This scoring method allows for objective classification of oncologists’ knowledge levels, enabling the identification of knowledge gaps and facilitating targeted training programs.

#### Data Analysis

Data analysis was conducted using SPSS (version 25), AMOS (version 26), and IRTPRO. Descriptive statistics were calculated for participant characteristics and item responses. EFA and CFA were conducted to validate the factor structure, and IRT analysis was used to evaluate item-level performance.

## Results

A total of 400 oncologists from cancer centers and oncology hospitals in Iraq participated in this study. The participants varied in their demographic and professional characteristics, ensuring a diverse and representative sample for the validation of the knowledge assessment tool.

The gender distribution of the participants showed that 60% (n = 240) were male, while 40% (n = 160) were female. In terms of educational background, most participants held either a Medical Doctor (45%) or Med Master (25%) qualification, followed by post-doctorate (17.5%) and Med Bachelor (12.5%) degrees, reflecting a highly qualified cohort.

Participants had a mean of 10.3 years (SD = 5.6) of professional experience as oncologists. Additionally, 70% (n = 280) of the oncologists reported having direct experience managing cases of radiotherapy resistance (RTR), which underscores the clinical relevance of the sample.

Regarding engagement in continuing education on RTR, the majority of participants (82.5%, n = 330) reported regularly or occasionally participating in professional development activities related to RTR, while 17.5% (n = 70) stated that they rarely or never engaged in such educational activities.

Access to resources on RTR was reported as easily accessible (55%) or moderately accessible (32.5%) for most participants. However, 12.5% (n = 50) of oncologists stated that RTR resources were difficult to access, indicating an area that may require attention to ensure accessibility of relevant materials Table 1.

**Table 1:** Sociodemographic characteristics of participants

Characteristic	Variable	*n* (%)
**Age (years)**	Mean (SD)	40.5 (8.2)
**Gender**	Male	240 (60%)
	Female	160 (40%)
**Educational Level**	Med Bachelor	50 (12.5%)
	Med Master	100 (25%)
	Medical Doctor	180 (45%)
	Post-doctorate	70 (17.5%)
**Years of Experience as an Oncologist**	Mean (SD)	10.3 (5.6)
**Experienced Radiotherapy Resistance Cases**	Yes	280 (70%)
	No	120 (30%)
**Engagement in Continuing Education on RTR**	Regularly	150 (37.5%)
	Occasionally	180 (45%)
	Rarely	50 (12.5%)
	Never	20 (5%)
**Collaboration with Other Specialists**	Yes	300 (75%)
	No	100 (25%)
**Accessibility of Resources on RTR**	Easily accessible	220 (55%)
	Moderately accessible	130 (32.5%)
	Difficult to access	50 (12.5%)
**Frequency of Radiotherapy Administration**	Daily	50 (12.5%)
	Weekly	100 (25%)
	Monthly	180 (45%)
	Occasionally	50 (12.5%)
	Rarely	20 (5%)

### Exploratory factor Analysis (EFA)

Table 77 presents the results of the Exploratory Factor Analysis (EFA) for the 29-item knowledge questionnaire on radiotherapy resistance (RTR). The analysis confirmed the presence of eight distinct factors, each representing a core knowledge domain. The factor loadings for all items were above 0.75, indicating strong alignment of items with their respective domains. Communality values ranged from 0.58 to 0.83, demonstrating that a substantial portion of each item's variance is explained by its corresponding factor.

The most significant findings highlight that the Sensitivity and Resistance domain showed the highest factor loadings, with the item on the role of the p53 pathway having a factor loading of 0.91, the strongest loading observed in the analysis. This suggests that oncologists'knowledge of the p53 pathway is a critical concept within this domain.

The Mechanisms of Action of Resistance domain also displayed strong loadings, with the item on genetic changes leading to resistance showing a high factor loading of 0.90. This reflects the conceptual importance of understanding how genetic changes drive resistance to radiotherapy, a key knowledge area for oncologists.

Other notable domains include Radioresistance and Limitations and Mechanisms of Radiation Therapy, which had consistently strong loadings for items related to the definition of radioresistance (0.83) and DNA double-strand breaks (0.87), respectively. These core concepts are fundamental to the understanding of radiotherapy resistance and are clearly captured within their respective domains Table 2.

**Table 2:** Exploratory Factor Analysis (EFA) Results of the 29-Item Knowledge Questionnaire

Domain	Item	Factor Loading	Communality
**Conventional and Combined Therapies**	1. Radiation therapy used alone or in combination	0.78	0.62
	2. Radiation effectiveness in metastatic stages	0.75	0.58
	3. Variability of radiation response across cancer types	0.80	0.64
**Radioresistance and Limitations**	4. Definition of radioresistance	0.83	0.70
	5. Resistance through repeated exposure to radiotherapy	0.85	0.72
	6. Role of genetic changes in radioresistance	0.82	0.68
	7. Types of radioresistance (innate, acquired, temporary)	0.88	0.75
**Mechanisms of Radiation Therapy**	8. Role of DNA double-strand breaks in cancer cell death	0.82	0.68
	9. Source of DNA damage in radiotherapy	0.85	0.72
	10. Cytotoxic effects of double-strand vs single-strand	0.87	0.76
	11. Intensity of radiation dose and double-strand breaks	0.84	0.70
**Sensitivity and Resistance**	12. DNA repair and cell sensitivity to radiotherapy	0.88	0.77
	13. Slowing of DNA repair in resistant cancer cells	0.91	0.83
	14. Epithelial tumor sensitivity vs lymphoid tumors	0.86	0.74
	15. Role of p53 pathway in radioresistance	0.85	0.72
**Radiosensitizers and Resistance**	16. Definition and role of radiosensitizers	0.80	0.64
	17. Use of cisplatin in non-small cell lung cancer	0.78	0.61
	18. Role of radicals in radiosensitization	0.82	0.67
**Chemotherapy and Radiation Resistance**	19. Resistance in chemotherapy and radiation	0.83	0.70
	20. Role of chemotherapy-radiation resistance in failure	0.85	0.72
	21. Cisplatin as a radiosensitizer in clinical practice	0.82	0.68
**Risk Factors**	22. Mutation rates and resistance development	0.87	0.76
	23. Advanced cancer stages and resistance	0.84	0.71
	24. Impact of prior chemotherapy on resistance	0.89	0.79
	25. Family history of cancer and resistance likelihood	0.86	0.74
**Mechanisms of Action of Resistance**	26. Genetic changes leading to resistance	0.90	0.81
	27. Changes in DNA repair mechanisms in cancer cells	0.88	0.78
	28. Cancer cell response to growth factors	0.85	0.72
	29. Tumor metabolism and dietary changes affecting response	0.87	0.76

### Confirmatory Factor Analysis (CFA)

The most significant findings from Table 3 highlight the items and domains with the highest factor loadings. Notably, the Sensitivity and Resistance in Cancer Cells domain had the highest factor loading of 0.91 for the item assessing oncologists'knowledge of the role of the p53 pathway in radioresistance. This strong loading reflects the critical role of the p53 pathway in cancer cell response to radiotherapy, making it a key determinant of knowledge in this domain.

**Table 3:** Construct Validity and Factor Loadings of the 29-Item Knowledge Questionnaire

Domain	Item	Factor Loading
**Conventional and Combined Therapies**	1. Radiation therapy used alone or in combination	0.78
	2. Radiation effectiveness in metastatic stages	0.72
	3. Variability of radiation response across cancer types	0.80
**Radioresistance and Limitations**	4. Definition of radioresistance	0.85
	5. Resistance through repeated exposure to radiotherapy	0.83
	6. Role of genetic changes in radioresistance	0.76
	7. Types of radioresistance (innate, acquired, temporary)	0.79
**Mechanisms of Radiation Therapy**	8. Role of DNA double-strand breaks in cancer cell death	0.84
	9. Source of DNA damage in radiotherapy	0.82
	10. Cytotoxic effects of double-strand vs single-strand	0.87
	11. Intensity of radiation dose and double-strand breaks	0.81
**Sensitivity and Resistance**	12. DNA repair and cell sensitivity to radiotherapy	0.88
	13. Slowing of DNA repair in resistant cancer cells	0.84
	14. Epithelial tumor sensitivity vs lymphoid tumors	0.79
	15. Role of p53 pathway in radioresistance	0.91
**Radiosensitizers and Resistance**	16. Definition and role of radiosensitizers	0.83
	17. Use of cisplatin in non-small cell lung cancer	0.80
	18. Role of radicals in radiosensitization	0.78
**Chemotherapy and Radiation Resistance**	19. Resistance in chemotherapy and radiation	0.85
	20. Role of chemotherapy-radiation resistance in failure	0.83
	21. Cisplatin as a radiosensitizer in clinical practice	0.87
**Risk Factors**	22. Mutation rates and resistance development	0.89
	23. Advanced cancer stages and resistance	0.82
	24. Impact of prior chemotherapy on resistance	0.84
	25. Family history of cancer and resistance likelihood	0.77
**Mechanisms of Action of Resistance**	26. Genetic changes leading to resistance	0.86
	27. Changes in DNA repair mechanisms in cancer cells	0.88
	28. Cancer cell response to growth factors	0.80
	29. Tumor metabolism and dietary changes affecting response	0.81

The Radioresistance and Limitations domain also demonstrated strong factor loadings, with the item on the definition of radioresistance achieving a factor loading of 0.85. This suggests that oncologists have a clear understanding of the concept of radioresistance, which is fundamental to the overall construct of the questionnaire.

In the Risk Factors domain, the item on the impact of mutation rates on resistance development had one of the highest factor loadings at 0.89, underscoring the importance of genetic mutations in influencing cancer resistance to radiotherapy. Similarly, in the Chemotherapy and Radiation Resistance domain, the item on the use of cisplatin as a radiosensitizer had a factor loading of 0.87, indicating oncologists'strong understanding of this clinically relevant concept.

### Item Response Theory (IRT)

The overall analysis of IRT shows that the difficulty (b) of items ranged from 0.68 to 1.25, indicating a mix of moderately challenging to difficult items. Items with the highest difficulty included"changes in DNA repair mechanisms"(b = 1.25) and"role of the p53 pathway"(b = 1.20), highlighting the complexity of these concepts for oncologists (Table 4). On the other hand, relatively easier items included"role of radicals in radiosensitization"(b = 0.68), which required a lower level of knowledge to answer correctly.

**Table 4:** Item Response Theory (IRT) Parameters for the 29-Item Knowledge Questionnaire

Domain	Item	Difficulty (b)	Discrimination (a)	Guessing (c)
**Conventional and Combined Therapies**	1. Radiation therapy used alone or in combination	0.85	1.25	0.10
	2. Radiation effectiveness in metastatic stages	0.72	1.30	0.12
	3. Variability of radiation response across cancer types	0.95	1.18	0.08
**Radioresistance and Limitations**	4. Definition of radioresistance	0.88	1.45	0.07
	5. Resistance through repeated exposure to radiotherapy	0.92	1.40	0.09
	6. Role of genetic changes in radioresistance	1.05	1.35	0.10
	7. Types of radioresistance (innate, acquired, temporary)	0.80	1.28	0.06
**Mechanisms of Radiation Therapy**	8. Role of DNA double-strand breaks in cancer cell death	1.10	1.50	0.05
	9. Source of DNA damage in radiotherapy	0.78	1.42	0.08
	10. Cytotoxic effects of double-strand vs single-strand	1.02	1.47	0.07
	11. Intensity of radiation dose and double-strand breaks	0.95	1.44	0.06
**Sensitivity and Resistance**	12. DNA repair and cell sensitivity to radiotherapy	0.70	1.60	0.04
	13. Slowing of DNA repair in resistant cancer cells	0.88	1.55	0.05
	14. Epithelial tumor sensitivity vs lymphoid tumors	1.02	1.33	0.06
	15. Role of p53 pathway in radioresistance	1.20	1.70	0.03
**Radiosensitizers and Resistance**	16. Definition and role of radiosensitizers	0.75	1.48	0.06
	17. Use of cisplatin in non-small cell lung cancer	0.92	1.50	0.07
	18. Role of radicals in radiosensitization	0.68	1.35	0.08
**Chemotherapy and Radiation Resistance**	19. Resistance in chemotherapy and radiation	1.15	1.40	0.06
	20. Role of chemotherapy-radiation resistance in failure	1.05	1.38	0.07
	21. Cisplatin as a radiosensitizer in clinical practice	0.90	1.50	0.05
**Risk Factors**	22. Mutation rates and resistance development	1.20	1.65	0.04
	23. Advanced cancer stages and resistance	0.95	1.45	0.05
	24. Impact of prior chemotherapy on resistance	0.85	1.42	0.06
	25. Family history of cancer and resistance likelihood	0.75	1.30	0.09
**Mechanisms of Action of Resistance**	26. Genetic changes leading to resistance	1.10	1.55	0.04
	27. Changes in DNA repair mechanisms in cancer cells	1.25	1.68	0.03
	28. Cancer cell response to growth factors	0.88	1.40	0.07
	29. Tumor metabolism and dietary changes affecting response	0.95	1.35	0.08

The discrimination (a) values ranged from 1.18 to 1.70, with the highest discrimination observed for the item on"role of the p53 pathway"(a = 1.70), indicating its strong ability to distinguish oncologists with high versus low levels of knowledge. Other high-discriminating items were"mutation rates and resistance development"(a = 1.65) and"changes in DNA repair mechanisms"(a = 1.68), reflecting their importance in differentiating participants with varying levels of understanding in these key areas of radiotherapy resistance.

The guessing (c) parameter values were low, ranging from 0.03 to 0.12, suggesting that the likelihood of oncologists correctly guessing the answers by chance was minimal. The lowest guessing parameter was observed for"changes in DNA repair mechanisms"(c = 0.03), indicating that this item required substantial knowledge to answer correctly.

### Reliability and Internal Consistency

The overall questionnaire had a Cronbach's alpha of 0.93 and a Composite Reliability (CR) of 0.94, indicating excellent internal consistency (Table 5). Among the eight domains, the Sensitivity and Resistance domain had the highest reliability, with a Cronbach's alpha of 0.90 and a CR of 0.91, reflecting the consistency of items assessing oncologists'knowledge of DNA repair, p53 pathways, and cancer cell resistance mechanisms.

**Table 5:** Reliability and Internal Consistency of the 29-Item Knowledge Questionnaire

Domain	Number of Items	Cronbach's Alpha	Composite Reliability (CR)
Conventional and Combined Therapies	3	0.82	0.84
Radioresistance and Limitations	4	0.85	0.87
Mechanisms of Radiation Therapy	4	0.88	0.89
Sensitivity and Resistance	4	0.90	0.91
Radiosensitizers and Resistance	3	0.84	0.85
Chemotherapy and Radiation Resistance	3	0.86	0.87
Risk Factors	4	0.89	0.90
Mechanisms of Action of Resistance	4	0.87	0.88
Overall Questionnaire	29	0.93	0.94

All domains exhibited strong reliability, with Cronbach's alpha values ranging from 0.82 to 0.90 and CR values ranging from 0.84 to 0.91, confirming that the items within each domain are internally consistent. These results demonstrate that the 29-item questionnaire is a robust, reliable tool for assessing oncologists'knowledge of RTR.

## Discussion

The present study aimed to develop and validate a comprehensive 29-item knowledge questionnaire to assess oncologists'understanding of radiotherapy resistance (RTR). The findings highlight the questionnaire's strong psychometric properties, including content, construct, and face validity, as well as reliability and item-level performance through IRT analysis. This study fills a crucial gap in the field of oncology, where the lack of standardized tools for assessing oncologists'knowledge of RTR has hindered efforts to enhance clinical decision-making and promote educational development in oncology care.

One of the key findings of this study is the validation of a 29-item questionnaire that covers eight distinct knowledge domains related to RTR. These domains include Conventional and Combined Therapies, Radioresistance and Limitations, Mechanisms of Radiation Therapy, Sensitivity and Resistance, Radiosensitizers and Resistance, Chemotherapy and Radiation Resistance, Risk Factors, and Mechanisms of Action of Resistance. The process of reducing the initial 45-item pool to 29 final items demonstrates a systematic and evidence-based approach to item refinement. Content and face validity were established through expert reviews, leading to the removal of items that lacked clarity, relevance, or appropriateness. This approach aligns with best practices in questionnaire development, as outlined by Zamanzadeh et al., who emphasize the role of expert review in achieving content validity [25]. In the context of Iraq, this tool addresses critical educational and clinical needs. Iraqi oncologists operate in a setting marked by limited access to advanced technologies, fragmented training in radiotherapy resistance, and infrastructure disparities across regions [5]. By enabling systematic knowledge assessment, this questionnaire can inform targeted CME (Continuing Medical Education) initiatives and support national efforts to standardize oncology education. Furthermore, it can guide curriculum development and strategic workforce planning in oncology, particularly as Iraq rebuilds its healthcare system post-conflict.

Exploratory Factor Analysis (EFA) confirmed the eight-domain structure of the questionnaire, with all factor loadings exceeding the 0.70 threshold, as recommended by Hair et al. for establishing construct validity [26]. The factor structure was further supported by Confirmatory Factor Analysis (CFA), where model fit indices (e.g., CFI = 0.92, TLI = 0.91, RMSEA = 0.06) demonstrated a good fit. These fit indices are consistent with established guidelines for structural equation modeling [27]. The reliability analysis showed that the overall questionnaire achieved a Cronbach's alpha of 0.93 and a Composite Reliability (CR) of 0.94, exceeding the commonly accepted threshold of 0.70 for internal consistency [28]. The IRT analysis confirmed the precision of individual items, with items exhibiting appropriate difficulty, discrimination, and guessing parameters, further supporting the robustness of the tool.

Furthermore, the development of knowledge assessment tools in the context of cancer resistance is relatively limited. Most existing tools have focused on general oncology knowledge or patient-related outcomes [29]. This study makes a novel contribution by focusing specifically on the unique and complex domain of radiotherapy resistance (RTR), which is rarely addressed in oncologists'training curricula. Previous research by Wang et al. [30] highlighted the need for specialized educational tools to bridge knowledge gaps in cancer resistance. Our study addresses this need by creating a questionnaire that captures the nuances of RTR knowledge.

Compared to similar tools developed in other healthcare domains, the internal consistency (Cronbach's alpha = 0.93) and Composite Reliability (CR = 0.94) observed in this study are notably high. For instance, tools assessing healthcare professionals'knowledge of antibiotic resistance have reported lower reliability values, typically ranging from 0.70 to 0.85 [31]. The higher reliability observed in this study may be attributed to the structured item development process and the focus on core theoretical concepts. Moreover, this study employed a rigorous validation process, incorporating Content Validity Index (CVI) and Modified Kappa Statistics, which are rarely used together in the development of knowledge tools [20]. This approach ensured that the retained items were not only relevant but also clear and free of bias.

### Strengths and Limitations

This study has several key strengths. First, the questionnaire development process adhered to established guidelines for content, face, and construct validity, using a multi-step process that incorporated expert input and psychometric analysis. The use of EFA, CFA, and IRT analysis ensured that only items with strong psychometric properties were retained, which strengthens the generalizability of the instrument.

Another strength is the sample of 400 oncologists from Iraq, a country with a strong network of oncology hospitals. This sample provides robust evidence for the instrument's applicability to oncologists practicing in low- and middle-income settings, where knowledge of cancer resistance is essential. The rigorous process of validation ensures that the questionnaire is suitable for broader international use.

However, several limitations should be noted. First, the study did not conduct a test–retest analysis, which is often used to evaluate the stability of scores over time. This may be addressed in future research. Finally, although the item pool was comprehensive, there is a risk that some concepts of RTR may not have been fully captured, especially as RTR mechanisms continue to evolve with advances in cancer biology.

## Conclusion

This study successfully developed and validated a 29-item knowledge questionnaire on radiotherapy resistance (RTR) for oncologists. The instrument demonstrated strong content, construct, and face validity, as well as high internal consistency (Cronbach's alpha = 0.93) and Composite Reliability (CR = 0.94). The use of IRT analysis confirmed that the items had appropriate difficulty, discrimination, and guessing parameters. The tool provides a reliable way to measure oncologists'knowledge of RTR, with significant implications for oncology education, clinical practice, and future research. By addressing an unmet need in the field, this study contributes a robust, theory-driven tool for assessing knowledge in a critical aspect of cancer care. Future research should focus on cross-cultural validation, test–retest reliability, and the application of the questionnaire to inform educational interventions and predict clinical outcomes. In Iraq specifically, this tool offers a foundation for evidence-based improvements in oncologist training, which may ultimately enhance the quality and consistency of patient care across the country’s oncology centers.

## Data Availability

Data and other materials are available upon request from the corresponding authors.
